# White Button Mushroom Extracts Modulate Hepatic Fibrosis Progression, Inflammation, and Oxidative Stress In Vitro and in *LDLR-/-* Mice

**DOI:** 10.3390/foods10081788

**Published:** 2021-08-01

**Authors:** Paloma Gallego, Amparo Luque-Sierra, Gonzalo Falcon, Pilar Carbonero, Lourdes Grande, Juan D. Bautista, Franz Martín, José A. Del Campo

**Affiliations:** 1 Unit for Clinical Management of Digestive Diseases and CIBERehd, Valme University Hospital, 41014 Seville, Spain; palgalyer@alum.us.es (P.G.); lourdes.grande.sspa@juntadeandalucia.es (L.G.); 2 Andalusian Center for Molecular Biology and Regenerative Medicine (CABIMER), University of Pablo de Olavide-University of Sevilla-CSIC, 41013 Seville, Spain; amparo.luque@cabimer.es; 3 Department of Biochemistry and Molecular Biology, Faculty of Pharmacy, University of Sevilla, 41012 Seville, Spain; falcong809@msn.com (G.F.); pcarboneroag@us.es (P.C.); jdbaut@us.es (J.D.B.); 4 Biomedical Research Network on Diabetes and Related Metabolic Diseases-CIBERDEM, 28029 Madrid, Spain; 5 ALGAENERGY S.A. Avda. de Europa 19, Alcobendas, 28018 Madrid, Spain

**Keywords:** atherosclerosis, hepatic fibrosis, high-fat diet, inflammation, mushroom extract, oxidative stress

## Abstract

Liver fibrosis can be caused by non-alcoholic steatohepatitis (NASH), among other conditions. We performed a study to analyze the effects of a nontoxic, water-soluble extract of the edible mushroom *Agaricus bisporus* (AB) as a potential inhibitor of fibrosis progression in vitro using human hepatic stellate cell (LX2) cultures and in vivo in *LDLR-/-* mice. Treatment of LX2 cells with the AB extract reduced the levels of fibrotic and oxidative-related markers and increased the levels of GATA4 expression. In *LDLR-/-* mice with high-fat diet (HFD)-induced liver fibrosis and inflammation, the progression of fibrosis, oxidative stress, inflammation, and apoptosis were prevented by AB extract treatment. Moreover, in the mouse model, AB extract could exert an antiatherogenic effect. These data suggest that AB mushroom extract seems to exert protective effects by alleviating inflammation and oxidative stress during the progression of liver fibrosis, possibly due to a decrease in Toll-like receptor 4 (*TLR4*) expression and a reduction in Nod-like receptor protein 3 (NLRP3) inflammasome activation. In addition, we observed a potential atheroprotective effect in our mouse model.

## 1. Introduction

Non-alcoholic fatty liver disease (NAFLD) is defined as the accumulation of fat in hepatocytes in the absence of excessive alcohol intake [1,2]. NAFLD is a widespread chronic liver disease that affects more than 30% of the Western global population [3,4] and is associated with many metabolic disorders, such as obesity, Type II diabetes, and cardiovascular diseases [5]. The histological spectrum of NAFLD begins with simple steatosis and oxidative stress [6]. Lipotoxicity triggers the hepatic inflammatory response, fibrosis progression, cirrhosis, and, ultimately, hepatocellular carcinoma [2].

Several families of pattern-recognition receptors (PRRs) recognize highly conserved pathogen-associated molecular patterns and host-derived damage-associated molecular patterns (DAMPs), and once activated, these receptors induce signaling cascades that ultimately lead to the induction of proinflammatory cytokines. Among these families are Toll-like receptors (TLRs) and Nod-like receptors (NLRs) [7,8]. A wide range of DAMPs can activate these PRRs, including obesity-related factors such as ceramide, fatty acids, hyperglycemia, mitochondrial dysfunction, and reactive oxygen species (ROS) [8]. In particular, saturated fatty acids (SFAs) can promote the development and progression of several noninfectious and inflammatory diseases, such as NAFLD, atherosclerosis, insulin resistance, and obesity, by activating Toll-like receptor 4 (TLR4) signaling in rodent models, promoting the inflammatory aspects of metabolic syndrome [7,9].

In particular, upon continuous liver inflammatory injury, TLR4 signaling is increased and enhances hepatic stellate cell (HSC) activation [10,11], leading to the release of fibrogenic mediators such as tropomyosin 2 (TPM2) [12], collagen (COL) types I, III, and IV and other contractile factors [13], several proinflammatory cytokines, and chemokines. All of these factors promote hepatic fibrosis [7] and inflammation through liver macrophage infiltration [14,15,16,17]. Conversely, the transcription factor GATA4 is produced by quiescent HSCs, regulates HSC inactivation, and promotes the suppression of fibrogenesis [18].

Moreover, obesity-associated DAMPs also activate multiprotein complexes through NLRs called inflammasomes, and the NLR family pyrin domain-containing 3 (NLRP3) inflammasome is the best characterized inflammasome [19]. Inflammasome activation leads to the cleavage, activation, and secretion of several proinflammatory cytokines, resulting in the modulation of the immune response [8].

Besides, emerging evidence has indicated that the NLRP3 inflammasome also participates in TLR4/NF-κB signaling pathway-mediated inflammatory response. Thus, inhibition of the TLR4/NF-κB/NLRP3 inflammasome pathway might be an effective approach to alleviating inflammation-related diseases [20,21].

Liver inflammation and ROS generation are also heightened by metabolic alterations in the β-oxidation pathway and fatty acid synthesis [22], whose balance is regulated by peroxisome proliferator-activated receptor α (PPARα), a transcription factor located in the nucleus that induces the expression of various genes involved in the β-oxidation pathway and is downregulated in NAFLD [19].

The oxidative stress plays a key role in a wide range of pathological processes, including chronic inflammation-related diseases and vascular disorders. The HFD-induced production of ROS is potentially toxic and can impair cellular or tissue integrity. Thus, antioxidant enzymes such as superoxide dismutase 1 (SOD1) and glutathione peroxidase 3 (GPX3) become activated to mitigate oxidative damage in hepatocytes [23,24]. The activation of inducible nitric oxide synthase (iNOS) contributes to local tissue destruction during chronic inflammation and oxidative processes by leading to the production of peroxynitrite, a strong physiological oxidant that activates liver immune cells and fibrogenesis [25,26,27], being thus another reliable marker related to oxidative stress.

Additionally, caspase-3 (CASP-3) is one of the most important downstream effector proteases in the nuclear changes associated with apoptotic cascades. Cleaved CASP-3 is able to result in hepatocyte apoptosis and eventually cause liver injury [28]. In HFD-induced NASH, apoptosis is also triggered by increasing effector caspases activation. The resulting apoptotic bodies are then phagocytized by adjacent cells, both Kuppfer cells and HSCs, which promotes inflammation and fibrogenesis [29].

*LDLR-/-* mice are susceptible to HFD-induced dyslipidemia, hepatic inflammation, and/or fibrosis that generally progresses to insulin resistance, which underlies the pathogenesis of NAFLD [30]. These risk factors are also implicated in the etiology and pathogenesis of cardiovascular diseases and atherosclerosis [31,32]. An HFD with 45% kcal from fats is sufficient to develop liver and cardiovascular system damage in this mouse model [33]. Compared to an HFD with 45% kcal from fat, an HFD (60% kcal from fat) can be more effective and quicker in inducing obesity, liver fibrosis, inflammation, and atherosclerosis lesions than the former [34,35,36,37].

The currently available drugs for treating liver injury can cause toxic side effects after long-term administration [38], and thus far, they have not presented an effective strategy against liver fibrosis in patients with NAFLD [39]. The white button mushroom AB shows antioxidant, immunomodulatory, antitumor, and hepatoprotective properties [40]. This food is considered by the Food and Agricultural Organization (FAO) to be a 21st century valuable health food [41] and true functional food [42]. Mushrooms or their extracts could be used as a potential treatment for liver diseases and have reduced toxicity. However, the bioactive compounds of this mushroom and the mechanisms of action have not yet been identified [43].

Our aim was to analyze the effect of an aqueous AB extract on LX2 cells to evaluate the ability of the extract to inhibit the progression of hepatic fibrosis and improve the oxidative stress-related markers. In addition, an in vivo study was performed using *LDLR-/-* mice fed an HFD (60% kcal coming from fats) to analyze the preventive effect of the AB extract against the progression of liver fibrosis and lipid accumulation in the aorta. We observed that AB extract exerted protective effects during the progression of liver fibrosis and hampered atherosclerosis development.

## 2. Materials and Methods

### 2.1. Preparation and Chemical Characterization of Natural Aqueous Mushroom Extract

AB was grown in a pilot plant at the University of Seville (Spain) according to standard procedures [44]. The characterization and fractionation of the crude extract was performed using sequential liquid–liquid extraction (Supplementary Materials). The extract enrichment process is shown in Table S1, the basic composition of AB extract is shown in Table S2, and the characterization results are shown in Table S3.

### 2.2. Cell Cultures

The LX2 cell line (human hepatic stellate cells) was acquired from Merck Millipore (SCC064, Burlington, MA, USA), and microscopic morphological analysis was performed to authenticate the cell line.

LX2 cells were seeded in 6-well plates at a density of 10^6^ cells per well with DMEM supplemented with 1000 mg/L D-glucose (Biowest, Nuaillé, France), 10% fetal bovine serum, 2 mM L-glutamine (Invitrogen, Madrid, Spain), 100 U/mL penicillin–streptomycin (Biowest), and 100× nonessential amino acids (Biowest). The cells were grown in an atmosphere of air/CO_2_ (95:5) with 90% humidity at 37 °C under sterile conditions.

A fluorescence microscopic assay was used for visual identification of mycoplasma infection in LX2 cell cultures using the MycoFluor Mycoplasma Detection Kit (Thermo Fisher, #M-7006, Waltham, MA, USA) according to the manufacturer’s recommendations.

To determine the nontoxic extract concentration for use on cells, a viability/toxicity assay was conducted in which cell morphology and growth were examined under the microscope using a variety of concentrations of the crude mushroom extract: 10, 5, 2, 1, and 0.2 mg/mL. Nontoxic concentrations were determined to be less than 2 mg/mL. The results are shown in Table S4.

Finally, LX2 cells were treated in five independent experiments with aqueous crude AB extract at concentrations of 0, 0.2, and 1 mg/mL for 24 h (*n = 5*).

### 2.3. Animal Diets and Experimental Design

Female LDL receptor-knockout mice (*LDLR-/-*, C57BL/6J background, *n = 19*, aged 16–17 weeks) were purchased from The Jackson Laboratory (Bar Harbor, ME, USA).

The mice were housed in a specific pathogen-free animal facility. Animal care and experimental protocols were in accordance with the guidelines for the Care and Use of Laboratory Animals of the Andalusian Center of Molecular Biology and Regenerative Medicine- CABIMER (Protocol number: 06-10-14-138). *LDLR-/-* mice are sensitive to energy-dense diets and develop obesity-associated pathologies, such as NAFLD [45,46,47]. However, there is not much information available about female mice. Thus, females were used in this study.

The mice were randomly divided into four groups at the beginning of the study and were allowed free access to diet and water. Two groups were fed a standard low-fat diet (LFD, 13 kcal% fat, 20 kcal% protein, and 67 kcal% carbohydrate, 11.8 kJ/g, Teklad Rodent Maintenance Diet 2014S, Harlan Laboratories, Indianapolis, IN, USA) without AB extract (LFD, *n = 5*) or with AB extract (LFD+AB, *n = 4*). The two other groups were fed an HFD based on lard (HFD-L). One diet contained a lower fat content (45 kcal% fat, 13% kcal% protein, and 41 kcal% carbohydrate, 15.7 kJ/g) without AB extract (HFD45, *n = 5*), and the other had a higher fat content to develop more severe damage (59 kcal% fat, 10 kcal% protein, and 31 kcal% carbohydrate, 21.0 kJ/g) plus AB extract (HFD60+AB, *n = 5*). The HFDs were prepared in our laboratory based on the LFD. The LFD and HFD45 groups constituted the negative and positive controls, respectively, while the LFD+AB and HFD60+AB groups were the treatment groups. Doses of AB extract were administered in the drinking water according to the weights of the mice (7.5 g/kg mice). The drinking water was changed three times per week. The average amount of AB extract that was consumed by the LFD group was 1.04 mg/mL (4.18 mg/cage/day) and by the HFD group was 1.29 mg/mL (6.43 mg/cage/day). To determine water (14.5 ± 4.4 mL/cage/day) and food intake (12.8 ± 3.5 g/cage/day), mice were placed, for one week, in the TSE Phenomaster monitoring system (TSE Systems GmbH, Bad Homburg, Germany). No significant differences were observed in water and food intake among the dietetic experimental groups (Figure S1).

Twelve weeks after the nutritional intervention, the mice were fasted for 12 h and sacrificed, and the liver and aorta were collected. The liver tissues were fixed for 12 h with 4% paraformaldehyde and paraffin embedded. The aortas were stored in PBS until en face preparation.

The composition of the experimental diets is shown in Table S5. A toxicity analysis of the effects of the extracts on the animals is shown in Table S6. Toxicity studies were performed on rats (Supplementary Materials). The studies indicated that in rats, the intake of AB extract at a dose that was 25 times higher than the average extract consumption in our mouse model did not show any acute toxicity.

### 2.4. Standard Biological Parameters

Mouse body weights (BWs) were measured, and blood samples were collected from the tail vein every 15 days during the 12 weeks of nutritional intervention. Mice were fasted for 12 h before blood collection. Blood glucose concentrations were measured using an automatic glucometer (Accu-Chek Aviva System, Roche, Indianapolis, IN, USA). Serum aspartate aminotransferase (GOT/AST) activity was analyzed with a Reflotron Plus system (Roche Diagnostics, Burgess Hill, West Sussex, UK) according to the manufacturer’s protocols.

### 2.5. Gene Expression Analysis

Total RNA was isolated from LX2 cells using TRIsure reagent (BIO-38033, Bioline Reagents Ltd., London, UK) and from mouse liver tissue homogenates using the RNeasy Lipid Tissue Kit (Qiagen, Hilden, Germany). RNA quantity was determined using a NanoDrop spectrophotometer (Thermo Scientific, Wilmington, DE, USA), and the quality was assessed with an Agilent 2100 Bioanalyzer (Agilent Technologies, Waldbronn, Germany). Reverse transcription was performed using a SensiFAST cDNA Synthesis Kit (Bioline, Memphis, TN, USA).

The expression of genes related to hepatic fibrosis (*COL1α1*, *ACTIN-2α*, *TPM2β*, and *GATA4*), oxidative stress (*SOD1*, *iNOS,* and *GPX3*), and steatosis and inflammation (*PPARα*, *TLR4,* and *TNFα*) was analyzed. Quantitative PCR was performed in triplicate for each sample using Eco Real-time PCR System v3.0 thermal cycler software (Illumina, San Diego, CA, USA, EEUU). The results are expressed as the relative gene expression normalized to the expression levels of the reference gene *GAPDH*. The primers are described in Tables S7 and S8 in the Supplementary Materials.

### 2.6. Western Blot Analysis

Cells were lysed, and protein levels were determined by SDS-PAGE coupled to Western blotting. Additional details for this protocol are provided in the Supplementary Materials. Antibodies were obtained commercially and included GATA4 (1:1000, Cell Signaling Technology, Danvers, MA, USA) and COL1α1 (1:1000, Aviva Systems Biology, San Diego, CA, USA). The secondary antibody was anti-rabbit (1:10,000, Santa Cruz Biotechnology Inc., Dallas, TX, USA). Image analysis was performed using Image Lab 6.0 software from Bio-Rad (Berkeley, CA, USA).

### 2.7. Liver Histology

After liver collection, fragments of four liver lobes of each mouse were paraffin-embedded (right, left, caudate, and quadrate lobes). Non-consecutive 6 µm slides were cut from the different lobes and three slides/mouse were used for histological analysis. Hematoxylin–eosin (H&E) and Sirius Red stains were used to evaluate pathological changes in the livers. The area of fibrosis, as determined by the deposition of COL1α1 fibers, was assessed by direct pixel counting of binary images captured by microscope at 10× magnification, by the same observer, and the average area was calculated from 10 randomly chosen fields per thin section for each animal. Immunofluorescence was performed to evaluate the presence of monocytes–macrophages, NLRP3 activation, and cleaved caspase-3-positive cells in the mouse liver, as previously described [48]. The primary antibodies used were anti-NLRP3 (1:100, LsBio, Seattle, WA, USA), anti-MOMA-2 (1:100, Abcam, Cambridge, UK), and anti-CASP-3 (1:400, Cell Signaling, MA, USA). The secondary antibodies were donkey CyTM3 anti-rabbit (1:200, Jackson, Cambridge, UK) for detecting NRLP3, Alexa Fluor 647 mouse anti-rat (1:300, Abcam, Cambridge, UK) for detecting MOMA-2, and goat Alexa Fluor 568 anti-rabbit (1:300, A-11011, Thermo Fisher, USA) for detecting cleaved CASP-3. The incubation with GATA4 was performed with the peroxidase substrate diaminobenzidine (DAB) to develop the color using the Vectastain Elite ABC kit (Vector Laboratories, Burlingame, CA, USA). The immunostaining was observed under bright-field light microscopy (Leica, Wetzlar, Germany). The analysis was conducted using ImageJ software (National Institutes of Health, Bethesda, MD, USA), and the results were normalized to the area of tissue that was observed.

### 2.8. Injury Analysis of the Aorta

Mouse aortas were isolated and fixed in 4% paraformaldehyde for 24 h. Next, to prepare en face sections of whole aortas, the attached adventitial tissues were removed, and the aortas were longitudinally opened and pinned onto black wax. Lipid accumulation in atherosclerotic lesions was quantified by staining with Oil Red O solution for 45 min and subsequently rinsing with isopropanol and distilled water. The percentages of lesions in the aortic arch were determined relative to the total luminal surface area of the aorta using ImageJ software (National Institutes of Health, Bethesda, MD, USA).

### 2.9. Statistical Analysis

Differences among pairs were tested using the unpaired Student’s *t*-test when a Kolmogorov–Smirnov test exhibited a normal distribution and when Levene F-test showed equal variances (parametric variables). When the variables were not normally distributed and/or the variances were not homogeneous, groups were compared using a Mann–Whitney U test (nonparametric variables). In order to test significant differences between groups considering two categorical independent variables (diets and time), the statistical analysis was performed using a two-way ANOVA. Post hoc tests were performed against HFD45 group using a Bonferroni test. All tests were two-tailed. GraphPad Prism 5.0 (La Jolla, CA, USA) was used to perform all analyses. All values are expressed as the means ± SD, and *p*-values less than 0.05 were considered statistically significant.

Moreover, a post hoc power analysis was performed using G*Power 3.1.9.2 software (Franz Faul, University of Kiel, Kiel, Germany), to determine effect size (*d*’Cohen) and the power (1-β err prob) of experimental diets. Effect size can be low (<0.2), medium (<0.5), and large (≥0.8).

## 3. Results

### 3.1. Treatment of LX2 Cells with AB Extract Reduced the Levels of Fibrotic and Oxidative Stress Markers and Increased the Levels of GATA4

**Figure 1:**
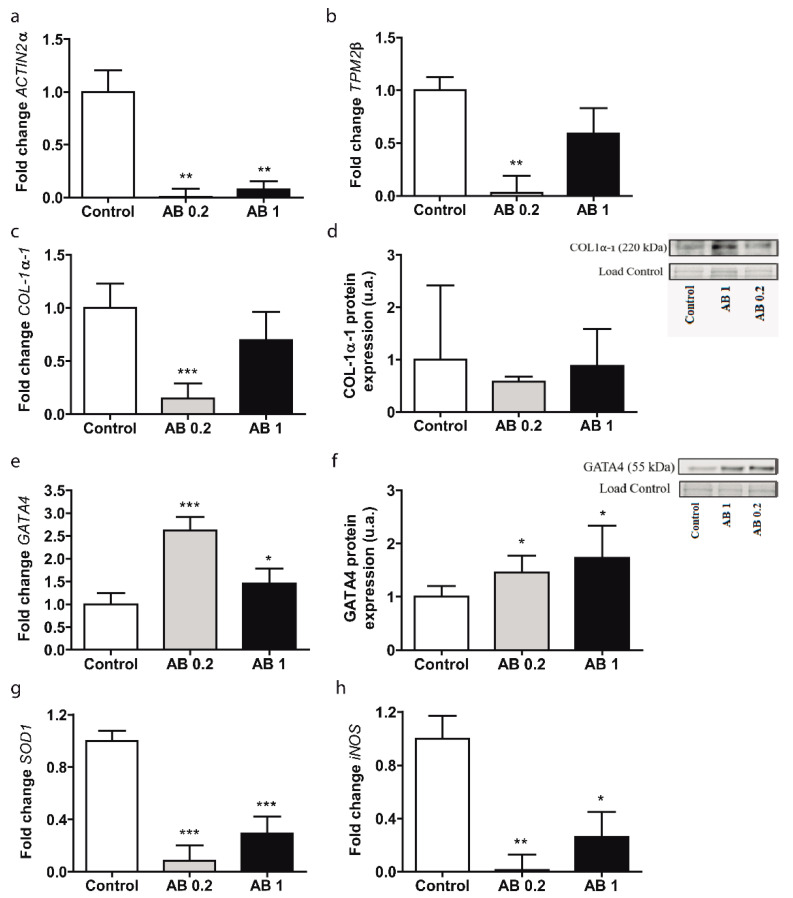
Gene expression levels of fibrotic and oxidative stress markers in LX2 cells treated with 0.2 mg/mL and 1 mg/mL AB extract for 24 h. (**a**) *ACTIN-2α*. (**b**) *TPM2β*. (**c**) *COL1α1*. (**d**) COL1α1 protein expression levels. (**e**) *GATA4*. (**f**) GATA4 protein expression levels. (**g**) *SOD1*. (**h**) *iNOS*. All data are shown as the means ± SD (*n =* 5). * *p* ≤ 0.05/** *p* ≤ 0.01/*** *p* ≤ 0.001 vs. the control.

*ACTIN-2α* levels were reduced after treatment with 0.2 and 1 mg/mL AB extract (*p* ≤ 0.01, Figure 1a) compared with those in control cells. *TPM2β* and *COL1α1* gene expression levels were reduced when LX2 cells were treated with 0.2 mg/mL AB extract (*p* ≤ 0.01, Figure 1b; *p* ≤ 0.001, Figure 1c, respectively), while no significant differences were found in response to 1 mg/mL compared to those in control cells. No significant changes were observed in the abundance of COL1α1 protein relative to that in control cells after exposure to both AB extract concentrations (Figure 1d). *GATA4* gene expression was significantly increased by 0.2 mg/mL (*p* ≤ 0.001) and 1 mg/mL (*p* ≤ 0.05) AB extract (Figure 1e) relative to that of control cells. The protein expression of GATA4 was significantly increased in the presence of different concentrations of AB extract (*p* ≤ 0.05, Figure 1f). Entire Western blots are shown in Figures S2 and S3.

*SOD1* gene expression was significantly decreased after treatment with both concentrations of AB (*p* ≤ 0.001, Figure 1g). The *iNOS* levels were significantly decreased by 1 mg/mL (*p* ≤ 0.05) and 0.2 mg/mL AB extract (*p* ≤ 0.01, Figure 1h).

### 3.2. AB Extract Inhibited the Increases in BW, Glycaemia, and Transaminase Levels in LDLR-/- Mice Fed an HFD Containing 60% Calories from Fats

**Figure 2:**
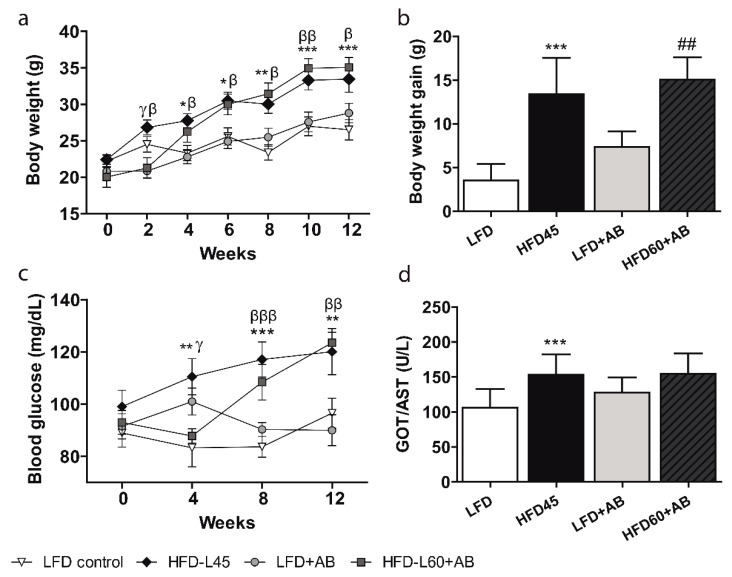
BW and biochemical blood parameters of *LDLR-/-* mice during 12 weeks of nutritional intervention with AB extract. (**a**) Changes in BW during the 12-week period. (**b**) BW gain at 12 weeks. (**c**) Glycaemia during the 12-week period. (**d**) Final GOT/AST levels. All data are shown as the means ± SD (*n =* 5). * p ≤ 0.05/** *p* ≤ 0.01/*** *p* ≤ 0.001 HFD45 vs. LFD; β *p ≤ 0.05/*ββ *p ≤ 0.01/*βββ *p* ≤ 0.001 HFD45 vs. LFD+AB; γ *p* ≤ 0.05 HFD45 vs. HFD60+AB; ## *p* ≤ 0.01 LFD+AB vs. HFD60+AB. The interaction of diet and time, with respect to body weight and glycemia, was analyzed by a two-way ANOVA.

The mouse BW changes throughout the 12-week period are reported in Figure 2a. Groups fed the HFD-L showed a significant increase in BW compared to the LFD groups (*p* ≤ 0.01 among control groups, *p* ≤ 0.05 among treated groups). Despite the higher fat and caloric content of the HFD60+AB diet (Table S5), the final BW values were similar in both HFD-L groups. The BW gain was significantly increased in the HFD-L groups compared to the LFD groups (*p* ≤ 0.05, Figure 2b). Again, no significant differences were observed in BW gain between the HFD groups, although the HFD60+AB group was fed a higher caloric diet. The blood glucose levels after 12 weeks were significantly increased in both HFD-L groups compared to the LFD groups (*p* ≤ 0.05 among the control groups, *p* ≤ 0.01 among the treated groups), but no differences were observed between the HFD groups, despite the higher fat and caloric content of the HFD60+AB diet (Figure 2c).

GOT/AST levels were significantly increased in the HFD45 group compared to the LFD group with large effect size (*p ≤ 0.001, d* = 1.679, *β* err = 0.011) after 12 weeks (Figure 2d). In contrast, no significant differences were observed between the AB-treated groups. Notably, despite the higher fat and caloric content of the HFD60+AB diet, these mice showed the same GOT/AST levels as HFD45 mice.

### 3.3. Liver Fibrosis, Inflammation, and Apoptosis Were Decreased in LDLR-/- Mice Treated with AB Extract after 12 Weeks of HFD Feeding

**Figure 3:**
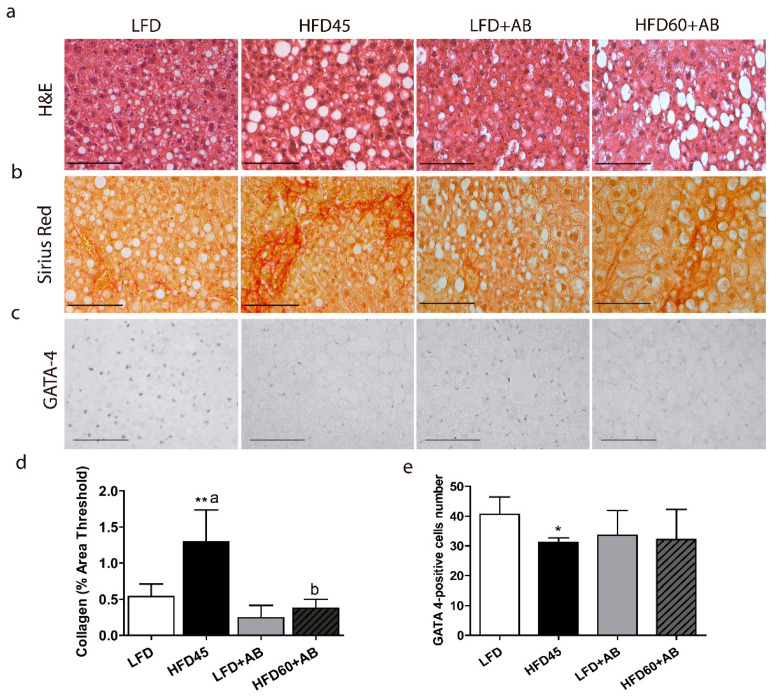
Histological analysis of liver injury induced by the HFD-L diets in *LDLR*-/- mice after 12 weeks of nutritional intervention with AB extract. Representative liver sections of (**a**) H&E staining, (**b**) Sirius Red staining, and (**c**) GATA4 HRP-immunostaining (40×, scale bar = 100 µm); (**d**) percent areas of hepatic collagen fibers stained with Sirius Red; (**e**) quantification of GATA4-positive cells. All data are shown as the means ± SD (*n* = 5). * *p* ≤ 0.05/** *p* ≤ 0.01 vs. LFD. Different letters indicate significant differences among the HFD groups (*p* ≤ 0.05).

H&E and Sirius Red staining showed the degree of injury to the liver (Figure 3a,b). In addition, liver GATA4-positive cells were also analyzed (Figure 3c). The HFD45 control group exhibited significant increases in fibrosis and a large effect size (*p* ≤ 0.01, *d* = 2.264, *β* err = 0.122) compared to those of the other groups. Notably, animals fed HFD60+AB showed the same level of fibrosis as those in the LFD and LFD+AB groups. These values were all significantly reduced (*p* ≤ 0.05, *d* = 2.834, *β* err = 0.026) with a large effect size compared to those of the HFD45 group (Figure 3d). Regarding GATA4, the HFD45 group showed significantly reduced levels compared to LFD with a large effect size (*p* ≤ 0.01, *d* = 2.198, *β* err = 0.017), while AB-treated groups did not differ with respect to LFD and HFD45 groups (Figure 3e).

**Figure 4:**
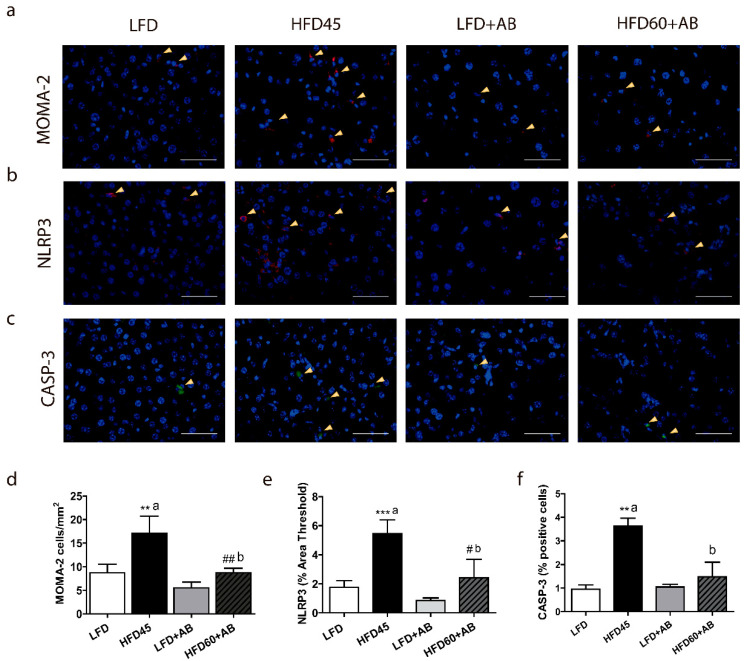
Histological analysis of liver inflammation and apoptosis induced by the HFD-L diets in *LDLR*-/- mice after 12 weeks of nutritional intervention with AB extract. Immunofluorescence showing (**a**) MOMA2 infiltration (red), (**b**) NLRP3-positive cells (red), and (**c**) CASP-3-positive cells (green). Nuclei were counterstained with DAPI (blue) (40×, scale bar = 100 µm). (**d**) Quantification of MOMA-2-positive cells. (**e**) Percent areas of NLRP3-positive cells. (**f**) Quantification of CASP-3-positive cells. All data are shown as the means ± SD (*n* = 5). ** *p* ≤ 0.01/*** *p* ≤ 0.001 vs. LFD; # *p* ≤ 0.05/## *p* ≤ 0.01 vs. LFD+AB. Different letters indicate significant differences among the HFD groups (*p* ≤ 0.05).

Inflammatory markers such as MOMA-2 and NLRP3 activation were assessed in the liver (Figure 4a,b). Moreover, apoptosis was studied, analyzing the percentage of liver CASP-3-positive cells (Figure 4c). 

The numbers of MOMA-2- and NLRP3-positive cells were significantly increased in the HFD45 control group compared to those in the other groups (*p* ≤ 0.01, *d* = 4.543, *β* err = 0.001). Notably, the HFD60+AB group had significantly fewer MOMA-2 infiltration (*p* ≤ 0.01, *d* = 3.655, *β* err = 0.008) and NLRP3-positive cells (*p* ≤ 0.05, *d* = 2.683, *β* err = 0.041) than the HFD45 group with a large effect size, despite the higher fat and caloric content of the diet in this group (Figure 4d,e). Finally, HFD45 significantly increased CASP-3 positive cells, compared to LFD (*p* ≤ 0.01, *d* = 9.922, *β* err = 0.000) and HFD60+AB groups (*p* ≤ 0.01, *d* = 4.308, *β* err = 0.001; Figure 4f) with a large effect size, indicating a possible antiapoptotic effect of AB extract despite the HFD intake.

### 3.4. AB Extract Reduced the Liver Expression of Genes Involved in Fibrosis, Oxidative Stress, and Inflammation in LDLR-/- Mice after 12 Weeks of HFD Feeding

**Figure 5:**
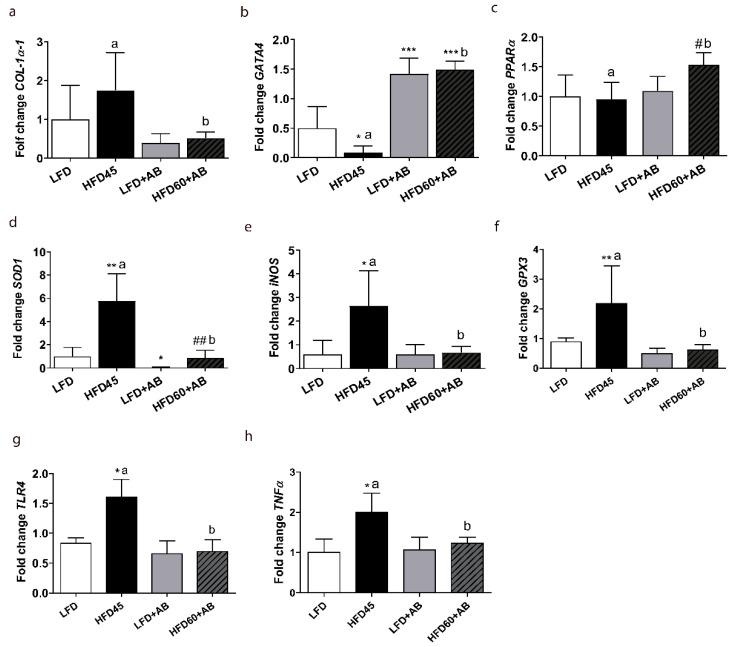
Gene expression analysis of fibrosis, oxidative stress, and inflammatory markers in the livers of *LDLR*-/- mice after 12 weeks of nutritional intervention with AB extract. (**a**) *COL1α1*. (**b**) *GATA4*. (**c**) *PPAR*α. (**d**) *SOD1*. (**e**) *iNOS*. (**f**) *GPX3*. (**g**) *TLR4.* (**h**) *TNFα*. All data are shown as the means ± SD (*n* = 5). * *p* ≤ 0.05/** *p* ≤ 0.01/*** *p* ≤ 0.001 vs. LFD; # *p* ≤ 0.05/## *p* ≤ 0.01 vs. LFD+AB. Different letters indicate significant differences among the HFD groups (*p* ≤ 0.05).

*COL1α1* gene expression levels of HFD45 were not significantly increased compared to LFD, but even so, produced a large effect size (*p* > 0.05, *d* = 0.803, *β* err = 0.798). Treatment with AB extract significantly reduced the levels of *COL1α1* in the HFD60+AB group compared to the HFD45 control group with a large effect size (*p* ≤ 0.05, *d* = 1.767, *β* err = 0.688, Figure 5a).

*GATA4* gene expression levels were significantly decreased in HFD45 compared to LFD with a large effect size (*p* ≤ 0.05, *d* = 1.515, *β* err = 0.443), and increased in both AB-treated groups compared to the LFD control group with a large effect size as well (*p* ≤ 0.001, *d* > 3.535, *β* err < 0.002). In addition, a significant increase and a very large effect size (*p* ≤ 0.001, *d* = 10.94, *β* err = 0.000) was observed in the HFD60+AB group compared to the HFD45 control group, despite the higher fat and caloric content (Figure 5b). HFD60+AB significantly increased *PPARα* expression levels compared with HFD45 (*p ≤* 0.01, *d* = 2.365, *β* err = 0.096), despite the higher fat and caloric content of this diet (Figure 5c). The HFD-L groups showed significant increases (*p* ≤ 0.01) in the expression levels of *SOD1* compared to those in their respective LFD groups. However, AB extract significantly reduced the levels of *SOD1* in both AB-treated groups vs. HFD45 group (*p* ≤ 0.05, *d* = 2.877, *β* err = 0.023; Figure 5d). The *iNOS* expression levels were significantly higher, with a large effect size (*p* ≤ 0.05, *d* > 1.8, *β* err < 0.277), in the HFD45 control group than in the other groups (Figure 5e). The gene expression level of *GPX3* was also significantly increased in HFD45 (*p* ≤ 0.01, *d* = 1.422, *β* err = 0.493; Figure 5f), while AB-treatment induced a significant reduction with a large effect size compared to HFD45 (*p* ≤ 0.01, *d* = 1.716, *β* err = 0.337). Moreover, we observed significant increases in *TLR4* levels in the HFD45 group (*p ≤* 0.05, *d* = 3.485, *β* err = 0.116) compared with the control LFD group. In addition, significant reductions with a large effect size in *TLR4* levels in the HFD60+AB group (*p ≤* 0.01, *d* = 3.603, *β* err = 0.098) were observed compared to those in the HFD45 group (Figure 5g). Finally, the TNFα gene expression level was also significantly increased by HFD45 (*p ≤* 0.01, *d* = 2.444, *β* err = 0.180, Figure 5h) but significantly reduced by HFD60+AB with a large effect size (*p ≤* 0.01, *d* = 2.202, *β* err = 0.262), despite its higher fat and caloric content. 

### 3.5. LDLR-/- Mice in the HFD60+AB Group Did Not Show More Aortic Atherosclerotic Lesions despite Higher Fat and Caloric Contents in Their Diet

**Figure 6:**
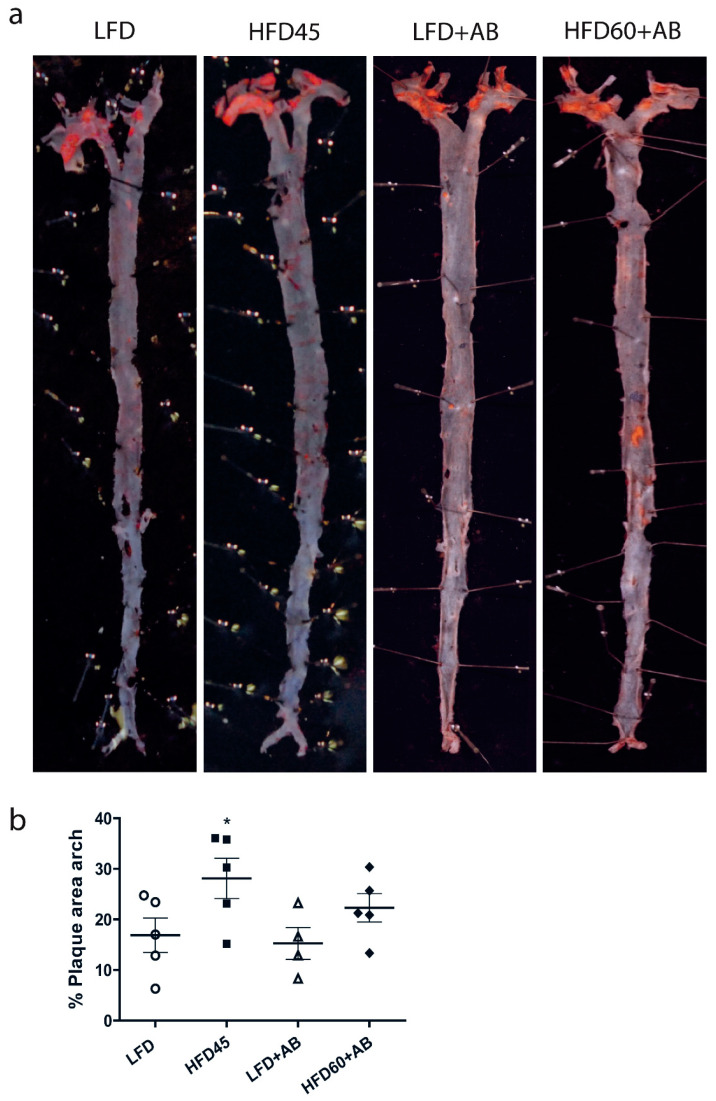
Analysis of atherosclerotic plaques in *LDLR*-/- mice after 12 weeks of nutritional intervention with AB extract. (**a**) Representative images of lesions in en face aortas stained with Oil Red O. (**b**) Aortic arch lesions in the different groups. All data are shown as the means ± SD (*n* = 5). * *p* ≤ 0.05 vs. the LFD control and LFD+AB groups.

The effects of AB treatment on the development of atherosclerotic plaques were assessed after 12 weeks of HFD feeding (Figure 6a). The atherosclerotic lesions were primarily distributed in the aortic arch. In the HFD45 control group, we detected a significant increase in the lesion area around the arch by 11.3% with a large effect size with respect to that in the LFD control group (*p* ≤ 0.05, *d* = 1.353, *β* err = 0.531). 

In addition, the HFD60+AB group did not significantly reduce its atherosclerotic lesions with respect to those in the HFD45 group, though it did produce a reduction with a medium effect size (*p* > 0.05, *d* = 0.749, *β* err = 0.818), despite the higher fat and caloric content of HFD60 (Figure 6b).

## 4. Discussion

Our aim was to investigate the possible antifibrotic, anti-inflammatory, and antioxidant effects of AB extract on LX2 cells, as well as in vivo, using mice with HFD-induced metabolic diseases [49]. The safety and tolerability of mushroom extracts have been tested in human subjects and animal models without adverse effects, and mushroom extracts are a possible natural product for the treatment of metabolic diseases such as obesity, diabetes, NAFLD, and atherosclerosis [50].

LX2 cells treated with 0.2 mg/mL AB extract exhibited significantly reduced *COL1α1*, *TPM2β,* and *ACTIN-2α* expression levels compared to those of the control, suggesting a significant antifibrotic effect of the AB extract. In another in vitro study using *Antrodia camphorate* extracts, which has a similar composition to our extracts, COL1α and III, ACTIN-2α, and fibronectin levels were decreased, possibly by inhibiting transforming growth factor β1 expression [51,52]. AB extract induced significant increases in GATA4 at the gene and protein expression levels, indicating the possible protective effect of the AB extract. We found better results at lower concentrations of AB (0.2 mg/mL) in most cell experiments. This finding can be explained by a hormetic effect [53,54].

Regarding our in vivo analysis, all mice with this genotype developed HFD-induced obesity. This effect was more evident in mice fed an HFD, but no significant differences in body weight were observed among the HFD45 (45% kcal fat) and HFD60+AB (60% kcal fat) groups, despite the higher fat and caloric content of the diet in the latter group. This result suggests that the AB extract could act as an adiposity-reducing agent that prevents BW gain. These data are supported by the findings of an in vivo study using HFD-fed ovariectomized mice supplemented with AB extract [55].

Serum GOT/AST, a biomarker related to hepatic damage [41], was not significantly increased in the HFD60+AB group compared to the LFD+AB group, despite the higher fat and caloric content in the diet of the former group. Nevertheless, significant increases in the GOT/AST levels were observed in the HFD45 group compared to the LFD control group. These data indicate a protective effect of dietary AB extract. In similar in vivo studies, reductions in serum transaminase levels were also found after treatment with dietary mushroom extracts [43,56].

Prolonged liver injury caused by an HFD leads to HSC and Kupffer cell activation associated with an increase in TLR expression, which results in the release of several proinflammatory mediators and abnormal liver extracellular matrix protein deposition [7,57]. 

In fact, under pathologic conditions as overnutrition, TLR signaling increases [58], while low mRNA levels of TLR4 are found in healthy livers, not activating TLR-signaling pathways [59].

In our study, the accumulation of collagen fibers in liver sections indicated a significant reduction in fibrosis in the AB-treated groups; in the same manner, *COL1α1* gene expression levels were significantly decreased in the hepatic tissues of mice with dietary AB extract, correlating with a decrease in *TLR4* expression. Moreover, the *GATA4* gene expression levels were significantly increased in AB-treated mice compared to those in their control groups, and GATA4-positive cells in liver sections did not differ from the LFD control group, so AB extract seems to protect against fibrosis by decreasing HSC activation, possibly due to a decrease in *TLR4* expression and an increase in *GATA4* expression.

Excessive nutrition causes an imbalance in hepatocellular metabolism mediated by hepatic mitochondria in NAFLD, which leads to an increase in mitochondrial respiration and the generation of ROS [50,60]. The overproduction of oxidative species induces the inflammatory response [61], and this response is accompanied by a reduction in antioxidant capacity [62], which promotes the activation of HSCs and liver damage. Other studies suggested a higher gene expression of antioxidant enzymes in response to a liver ROS overproduction, in order to counteract the oxidative stress [63,64]. Besides, oxidative stress-induced hepatic damage is also affected by imbalance of other enzymes, such as iNOS, whose gene levels are highly expressed in HFD-induced NASH models [65]. This could be happening in livers from mice of the HFD45 group, whose diet induced a significant increase of *SOD1*, *GPX3,* and *iNOS* gene levels, while in both AB-treated groups, a significant decrease in those gene expression levels were observed. This could indicate a reduced compensatory mechanism. Thus, the above data suggests that mushroom extracts could have antioxidant effects on LX2 cells and in *LDLR-/-* mice. Previous in vivo studies have shown the antioxidant effects of mushroom extracts, including AB extracts [66,67]. 

AB mushroom has a high fiber, but low fat and cholesterol content [68]. Besides, it has a high content of bioactive compounds such as β-glucans, phenolic compounds, terpenes, β-carotenes, minerals, and important vitamins. All these compounds are related to its antiviral, antioxidant, and anti-inflammatory properties [69]. Different studies have positioned *Agaricus bisporus* as an antioxidant therapeutic option for a wide variety of diseases, including immune and inflammatory disorders, cancer, hypertension, diabetes, and hyperlipidemia, because of its phenolic composition [70,71]. 

Its bioactive compounds seem to regulate and improve the antioxidant defense system, maintaining the oxidative homeostasis [72]. Another study also showed that the use of extracts from edible mushrooms, as a supplement in the diet, decreased the plasma levels of aminotransferases, free radicals, glutathione peroxidase, and superoxide dismutase [73]. Another recent study, using pigs fed AB mushrooms for 6 weeks, showed reduced levels of inflammatory cytokines involved in the activation of the NLRP3 inflammasome complex [74]. The immunomodulatory and antioxidant activities of most fungal bioactive compounds seem to be attributed to chitin or β-glucans [75] found in ergothioneine and selenium-enriched mushrooms [76], and these compounds are found in the AB extract. In particular, the ergothioneine is one of the most potent bioactive compounds of AB extract, which shows a protection role against mitochondrial DNA damage caused by superoxide anion generation [77,78]. It is present in raw extracts of the genus Agaricus, with similar composition to the genus *Pleurotus*, capable of reducing the lipid hydroperoxide formation and protecting against polyunsaturated fatty acids degradation [79]. 

Mitochondrial dysfunction and increased stress signals promote the infiltration of monocytes and macrophages in the liver, which encourages the progression of chronic liver injury and fibrosis through HSC activation and proliferation [80] and through the activation of caspase-1 and IL-1β via the NLRP3 inflammasome [8]. TLR4 may also play an important role in the promotion of macrophage infiltration and inflammatory cytokines secretion by activating inflammatory signaling pathways such as JNK and NF-κB, and thereby aggravating liver damage [81]. In turn, low expression of TLR4 by hepatic dendritic cells may contribute to the reduced or altered activation of hepatic adaptive immune responses [59]. Previous studies have shown HFD-stimulated hepatic infiltration of macrophages in *LDLR*-KO mice, but these effects were abolished by TLR4 deficiency and decreasing the expression of important components of the NLRP3 inflammasome [19,82]. Thus, liver integrity and inflammatory processes might be associated with a decrease in the expression of TLR4 and Nod signaling-related genes [83]. In our study, no severe inflammatory response was observed in the HFD60+AB group, as reflected by the twofold decrease in MOMA2-positive cells compared to those in the control groups. Additionally, the NLRP3 inflammasome was significantly less activated in the AB-treated groups than in both control groups, showing a correlation with lower *TNFα* gene expression levels. This finding indicates that the dietary AB extract could have anti-inflammatory activity. Another study, using diabetic mice fed mushroom extract, showed alleviation of NAFLD through decreased inflammatory damage by suppressing proinflammatory MCP-1 production [23]. From the above results, we propose that AB extract could inhibit the TLR4/NF-κB/NLRP3 inflammasome pathway. This would reduce inflammation and oxidative stress, which in turn could increase the resistance to HFD-induced liver injury. 

Regarding liver apoptotic processes, caspases are proteases that stimulate apoptosis in damaged cells [84]. Our data showed that AB treatment significantly lowered the contents of liver cleaved-CASP-3 positive cells, suggesting its ability to ameliorate HFD-induced apoptosis.

In addition, previous studies have reported that AB extract may improve the atherogenic index and reduce cardiovascular risk in diabetic rats [43], but there have been no studies regarding its antiatherogenic effects on mouse models with diet-induced liver damage. In our study we have shown, for the first time, the potential capacity of dietary AB extract to prevent the exacerbation of atherosclerotic lesions in HFD-fed models, since no differences of lesion sizes were observed between the LFD and HFD-L groups that were administered AB extract, despite the higher fat and caloric content in the HFD60+AB group compared to the HFD45 group. In the absence of further studies, this finding suggests the potential atheroprotective effect of AB extract on diet-induced liver damage mouse models. Additional analysis of atherosclerosis markers is needed to complete this study and to unravel the mechanisms behind this effect.

**Figure 7:**
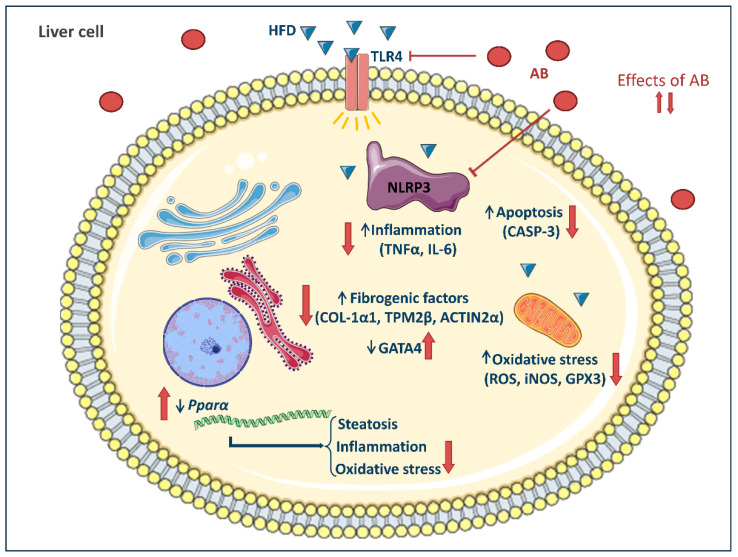
Potential mechanisms of action of AB bioactive compounds on inflammation and oxidative stress processes. The saturated fatty acids of the HFD and/or adipose tissue lipolysis enter the bloodstream and act as ligands of the TLR4 receptor. This binding activates several signaling pathways in liver cells to promote the progression of liver damage. AB extract promotes a decrease in TLR4 activation and oxidative stress production in the cell, which reduces NLRP3 inflammasome activation and apoptotic markers, increases *Pparα* mRNA expression, and decreases the gene expression of fibrogenic molecules, reducing inflammation, oxidative stress, and fibrosis.

In conclusion, our study shows that aqueous mushroom extracts attenuate liver damage in LX2 cells and in HFD-fed *LDLR-/-* mice through the alleviation of inflammation and oxidative stress-related markers, possibly due to AB extracts acting directly or indirectly as inhibitors of *TLR4* expression and NLRP3 inflammasome activation (Figure 7).

AB extract also prevented the progression of atherosclerotic lesions. These results support the recommendation of consuming common mushrooms as an inexpensive, nontoxic, and preventive alternative to lessen the progression of Western diet-induced liver damage to liver fibrosis. Nutritional studies are necessary to analyze the potential benefits of dietary mushroom extracts in carefully controlled clinical trials, and further work is needed to reveal the molecular mechanisms that are involved in their protective effects.

## Data Availability

The data presented in this study are available on request from the corresponding author.

## Supplementary Materials

The following are available online at https://www.mdpi.com/article/10.3390/foods10081788/s1. Table S1. Result of the processing of the variety of fungi (*A. bisporus*). Table S2. Basic composition of AB extract. Table S3. List of metabolites identified in the different fractions of AB extract. Table S4. Percentage of LX2 cells death using different concentrations of AB mushroom extract. Table S5. Macronutrient composition of experimental diets. Table S6.a. Distribution of control and treated groups for acute toxicity study in rats. Table S6.b. Acute toxicity study in rats (dose 5000 mg/kg BW of AB extract). Table S7. List of specific human primers. Table S8. List of specific mouse primers. Figure S1. Indirect calorimetric analyses of (a) average food intake mouse/day and (b) average drink intake mouse/day. Figure S2. Blot images (*n* = 5) of protein expression experiments in LX2 cells treated with AB extracts at 0.2 mg/mL and 1 mg/mL for 24h. Figure S3. Blot images (*n* = 5) of protein expression experiments in LX2 cells treated with AB extracts at 0.2 mg/mL and 1 mg/mL for 24 h [85,86,87,88,89].

## Abbreviations

AB (*Agaricus bisporus*), BW (body weight), CASP-3 (caspase-3), COL (collagen), DAMPs (damage-associated molecular patterns), GATA4 (GATA Binding Protein 4), GOT/AST (aspartate aminotransferase), GPX3 (glutathione peroxidase 3), H&E (hematoxylin-eosin), HFD (high-fat diet), HSCs (hepatic stellate cells), iNOS (inducible nitric oxide synthase), LDLR (LDL-receptor), LFD (low-fat diet), MOMA-2 (monocytes and macrophages), LX2 (human hepatic stellate cells), NAFLD (nonalcoholic fatty liver disease), NASH (nonalcoholic steatohepatitis), NLRs (Nod-like receptors), NLRP3 (Nod-like receptor protein 3 inflammasome), PPARα (peroxisome proliferator-activated receptor alpha), PRRs (pattern-recognition receptors), ROS (reactive oxygen species), SOD (superoxide dismutase), TLRs (Toll-like receptors), TLR4 (Toll-like receptor 4), TPM2 (tropomyosin 2).
