# Supplementary material for: White Button Mushroom Extracts Modulate Hepatic Fibrosis Progression, Inflammation, and Oxidative Stress In Vitro and in LDLR-/- Mice
Source: Foods. 2021 Aug 1;10(8):1788. doi: 10.3390/foods10081788 (PMC8392037; doi:10.3390/foods10081788)
Supplement: Supplementary file 1 [file foods-10-01788-s001.zip › foods-1210211-supplementary update .pdf]

White button mushroom extracts modulate hepatic fibrosis progression, inflammation and oxidative stress *in vitro* and in *LDLR*-/- mice.

Paloma Gallego[1†], Amparo Luque-Sierra[2†], Gonzalo Falcon[3], Pilar Carbonero[3], Lourdes Grande[1], Juan D Bautista[3], Franz Martín[2,4*], José A del Campo[1*].

Supplementary Materials

Composition of *Agaricus Bisporus* (AB) Extract

Basic composition of *Agaricus bisporus* (AB) extract is shown in Tables S1 and S2. The majority fraction of the dry weight of edible mushrooms is that corresponding to carbohydrates. The carbohydrates present in edible mushroom are found in the form of monosaccharides, disaccharides, oligosaccharides, and polysaccharides. Polysaccharides include glucans (α-D-Glucans and / or β-D-glucans, chitin and mannan Although most of the carbohydrates in edible mushrooms are in the form of polysaccharides (glucans, chitin, and glycogen), edible mushrooms also contain other simple carbohydrates such as pannitol, which can represent up to 30% of dry weight .

The second largest fraction in edible mushrooms is made up of protein compounds: amino acids, peptides, and proteins. Mushrooms contain practically all the essential amino acidsfor humans: histidine, isoleucine, leucine, lysine, methionine, phenylalanine, threonine, tryptophan, and valine. Despite its low-calorie content, the protein content is equivalent to that of some legumes, and its composition is rich in essential amino acids, providing significantly more tyrosine, phenylalanine, threonine, cysteine, and lysine than vegetable such as carrots, cauliflower, or potatoes. In addition, edible mushrooms also contain other non- proteogenic amino acids, such as ergothioneine with antioxidant properties beneficial to health.

Although edible mushrooms are products with a low-fat content, this is made up of free fatty acids, mono-, di- and triglycerides, sterols, tocopherols, and phospholipids.

Preparation and chemical characterization of natural aqueous mushroom extract

Briefly, mushrooms homogenization was performed (10 g + 10 mL distilled water), followed by enzymatic digestion with a mixture of glucanase and chitinase enzymes (Novo Nordisk, Madrid, Spain) at pH= 5.0 and an enzyme/substrate ratio of 0.01 at a temperature of 55ºC for 24h. After this, the temperature was raised to 90ºC for 120 min to inactive the enzymes. After cooling to room temperature, the pH was adjusted to 7.0 with 1 M NaOH and centrifuged at 8000 x *g*. The supernatant was collected and filtered through a 0.2 µm membrane to obtain the crude aqueous extract.

For characterization, fractionation of AB crude extract was performed by sequential liquid-liquid extraction using three solvents (hexane, dichloromethane and ethylacetate). Thetwo-phase system obtained was centrifuged at 1500 × *g* for 5 min, and each of the phases were collected. Then, 5 mL of each extract (hexane, dichloromethane, ethyl acetate and aqueous phase) were concentrated to 0.5 mL in the speed-vac and then freeze-dried using a Telstar lyophilizer (Barcelona, Spain). Identification of mushroom compounds was conducted with a liquid chromatography quadrupole time-of-flight mass spectrometry (LC-QTOF MS/MS, Agilent 6540 UHD Accurate-Mass QTOF, Santa Clara, CA, USA) analysis in high-resolution mode. The analytical samples were injected in LC-QTOF and in an auto MS/MS system to obtain information from the compounds fragmentation. The complexity of the mushroom extract required the selection of extractants with different polarity indexes to enhance the compound identification. The lists of metabolites identified in the different fractions of the AB extract are shown in Table S3.

Determination AB extract toxicity in rats

An acute toxicity test was conducted to evaluate any possible adverse effects that could occur following the oral administration of a single dose of AB extract. For this study, the acute toxic class method was used. This procedure is reproducible and uses very few animals. This method is based on biometric evaluations with fixed doses that are adequately separated to enable a substance to be ranked for classification purposes and for hazard assessment. Wistar rats were bred and characterized in Charles River laboratories (Écully, France). Rats were housed in a specific pathogen-free animal facility. Animal care and experimental protocols wereadjusted to follow the guidelines for the care and use of laboratory animals of the Faculty of Pharmacy (University of Seville). Forty rats were randomly divided into four groups: one control group (ACT-0) and three treated groups (ACT I-to-III). Each group was composed of 10rats, 5 per sex (**See Table S6a**). After fasting for 12 h, rats were orally administered (by gavage)a single dose of 300, 2000 or 5000 mg/kg body weight (BW) of AB extract dissolved in 3 mL ofdistilled water; the control group was treated with distilled water (3 mL). After administration ofthe preset doses, rats were allowed access to food and water *ad libitum*, and behavior changes, toxic symptoms, and deaths were continuously observed for 1 h after treatment and thenintermittently at 4, 8 and 24 h. After these initial observations, rats were further observed for up to 14 consecutive days for any signs of toxicity and/or death. Any adverse effects, such as hypoactivity, fur-erection, salivation, and syncope were evaluated after the administration of thedifferent doses of AB extract. BW were measured on day 1, 7 and 14. Food and water intake were registered every two days, and anorexia and weight loss were recorded. All tested animals were subjected to gross necropsy and microscopic examination. **Table S6b** shows the effectsand general behavioral changes of rats following oral administration of AB extract at the 5000 mg/kg doses. Since no death was observed at this dose, the LD_{50} could not be estimated but is expected to exceed 5000 mg/kg. Therefore, it can be concluded that the acute toxicity of AB extract by the oral route can be classified as category 5 or even unclassified in the Globally Harmonized System of Classification and Labelling of Chemicals (GHS). In conclusion, the

absence of signs of morbidity and mortality in the oral dose study provide evidence to support the safety of the short-term oral administration of AB extract.

Western blot analysis

Cells were treated with lysis solution that included 50 mM HEPES pH 7.5, 5 mM EDTA, 150 mM NaCl, 1% 4-nonylphenyl-polyethylene-glycol, commercial protease inhibitor cocktail (P8340, Sigma-Aldrich, Madrid, Spain), 1 mM phenylmethylsulfonyl fluoride, 1 mM NaF and 1 mM Na_3VO_4. Proteins (50-100 µg) were separated by an Any kD™ Criterion™ TGXStain-Free™ Protein Gel, 18 well, 30 µL (#5678124, BioRad, Hercules, CA, USA) and transferred to PVDF membranes. The system uses stain-free technology, which is a method that appears to be more reliable as a protein loading control than the measurement of housekeeping proteins. The membranes were incubated with the corresponding commercial primary and secondary antibodies that were coupled to horseradish peroxidase to reveal the protein content by the Clarity™ Western ECL substrate (Ref 170-5061, BioRad) and were analyzed in a ChemiDoc Touch Imaging System (BioRad).

Table S1. Result of the processing of the variety of fungi *A. bisporus*. Initial weight (g) of fresh matter, final weight (g) after drying, % dry weight and volume of H_2O_d (mL) required for a concentration of 42 mg/mL.

Mushroom species	Initial weigth (g)	Final weight (g)	Dry weigth (%)	H_2O_d (mL)
A.bisporus	12.91	0.37	2.86	8.81

Table S2. Basic composition of AB extract. Composition data of dry matter and moisture are expressed in % on the total weight of the mushroom fruiting body. Data of AB extract chemical composition is expressed in % Dry Matter ± SD. Protein-N (protein nitrogen), which belongs to free amino acids (Free AAs), oligopeptides or peptides; total carbohydrates; crude fat which is not detected and other compounds(Others) were calculated on the average value of the rest of the compounds. Energy (Kcal/100 mg wet weight).

Mushroom Extract	*Agaricus bisporus*
**Dry Matter (%)**	7.20 ± 4.12
**Moisture (%)**	92.80 ± 0.54
**Proteín-N (% Dry Matter)**	27.17 ± 2.98
**Free AAs**	3,85
**Oligopeptides < 5 kDa**	20,12
**Peptides > 5 kDa**	3,20
**Total Carbohydrates (% Dry Matter)**	41.63 ± 3.22
**β-Glucans (% D.W.)**	4,32 ± 0,34
**Crude fat (% Dry Matter)**	n.d. (not detectable)
**Others**	15,89
**Energy (Kcal/ 100 g W.W)**	37,1 ± 2.2

Table S3. List of metabolites identified in the different fractions of AB extract.

Metabolite	MS^1 spectra (m/z)	Product ions (m/z)	AB extract	Delgado-Povedano *et al.*, 2016
**Alkaloid derivative**				
**2-Methylpyrrolidine**	86.0969	43.0539<br>41.0390	+	+
**Alkylamines**				
**Pantothenic acid**	220.1178	90.0556<br>72.0443	+	+
**Spermidine**	146.1651	84.0811<br>72.0809	+	+
**Pyrroline**				
**1-Pyrroline**	70.0652	43.0542<br>41.0385	+	+
**Amino acids and derivatives**				
**2,5-Dihydrophenyl-alanine**	168.1019	126.0551<br>81.0707	-	+
**2-Aminobutyric acid**	102.0562	102.0562	+	+
**3-Amino-2-naphthoic acid**	188.0703	170.0594<br>142.0651	+	+
**3-Amino-3-(4-hydroxyphenyl)propanoate**	180.0665	119.0495<br>93.0340	+	+
**5-Aminopentanoate**	118.0864	74.0588<br>58.0625	+	+
**Acetylcarnitine**	204.1231	85.0288<br>60.0809	+	+
**Citrulline**	176.1031	113.0708<br>70.0650	+	+
**Cycloleucine**	130.0863	84.0809<br>67.0540	+	+
**Cystathionine**	223.0746	134.0267<br>88.0212	+	+
**Ergothioneine**	230.0957	127.0322<br>60.0809	+	+
**L-2-Aminoadipic acid**	160.0616	116.0720<br>98.0609	+	+
**L-Agaritine**	268.1292	84.0439<br>77.0389	+	+
**L-Arginine**	173.1040	131.0823<br>112.0868	+	+
**L-Asparagine**	133.0610	74.0233<br>46.0283	+	+
**L-Aspartic acid**	132.0302	88.0405<br>71.0139	+	+
**L-Carnitine**	162.1124	103.0389<br>60.0808	+	+
**L-Glutamate**	148.0602	85.0284<br>56.0494	+	+
**L-Glutamine**	147.0761	84.0442<br>56.0493	+	+

**L-Isoleucine**	132.1019	86.0967<br>69.0699	+	+
**LL-2,6-Diaminoheptanedioate**	189.0886	84.0444<br>74.0237	+	+
**L-Leucine**	132.1020	86.0967<br>44.0492	+	+
**L-Lysine**	147.1129	84.0809<br>56.0492	-	+
**L-Methionine**	150.0585	61.0105<br>56.0496	+	+
**L-Phenylalanine**	166.0860	120.0804<br>103.0544	+	+
**L-Proline**	116.0703	70.0652<br>43.0539	+	+
**L-Serine**	104.0353	74.0243<br>42.0356	+	+
**L-Threonine**	120.0651	74.0599<br>56.0495	+	+
**L-Tryptophan**	205.0972	146.0600<br>118.0649	+	+
**L-Tyrosine**	182.0806	136.0753<br>91.0542	+	+
**L-Valine**	118.0863	72.0809<br>55.0543	+	+
**N2-Acetyl-L-ornithine**	175.1078	116.0692<br>70.0653	+	+
**N6-Acetyl-L-2,6-diaminoheptanedioate**	233.1134	87.0433<br>84.0465	+	+
**N-Carbamylglutamate**	189.0518	128.0347<br>102.0560	+	+
**Nε-Acetyl-L-lysine**	189.1230	126.0909<br>84.0811	+	+
**Ornithine**	133.0973	116.0708<br>70.0651	-	+
**Pipecolic acid**	130.0864	84.0810<br>56.0493	+	+
**Pyroglutamic acid**	128.0353	128.0352<br>82.0294	+	+
**Saccharopine**	277.1393	130.0864<br>84.0809	+	+
**Peptides**				
**Cyclo(L-phe-L-pro)**	245.1286	120.0802<br>70.0654	-	+
**Oxidized glutathione**	611.1446	306.0762<br>272.0886	+	+
**γ-D-Glutamylglycine**	205.0822	142.0494<br>84.0442	+	+
**Quinoline derivative**				
**5,6-Dihydroxy-3-methyl-2-oxo-1,2,5,6-tetrahydroquinoline**	194.0812	120.0455<br>57.0332	-	+
**Indoles**				
**Formyl índole**	146.0596	118.0649	-	+

		91.0543		
**Indole-3-acetate**	174.0559	144.0439<br>130.0656	-	+
**Benzaldehyde**				
**4-Hydroxybenzaldehyde**	123.0444	95.0484<br>77.0381	+	+
**Carboxylic acids and derivatives**				
**2,2-Dymethyl succinic acid**	145.0505	101.0602<br>83.0504	+	+
**3-Hydroxy-3-methyl-glutaric acid**	161.0454	59.0143<br>57.0349	+	+
**Citric acid**	191.0195	111.0093<br>87.0089	+	+
**Lactic acid**	89.0246	43.0188<br>41.0032	+	+
**Malic acid**	133.0142	72.9928<br>71.0135	+	+
**Succinic acid**	117.0198	73.0297<br>55.0194	-	+
**Cinnamic acid derivative**				
**p-Coumaric acid**	165.0542	147.0442<br>119.0493	+	+
**Disaccharides**				
**Cellobionic acid**	357.1043	113.0233<br>89.0233	+	+
***α*-1,5-L-Arabinobiose**	281.0876	89.0244<br>71.0132	+	+
**Monosaccharides**				
**Sedoheptulose-7-phosphate**	289.0332	96.9693<br>78.9592	+	+
**D-Glucosamine 6-phosphate**	258.0385	171.0060<br>78.9615	-	+
**D-Glucose 6-phosphate**	259.0226	96.9693<br>78.9592	+	+
**D-Mannitol**	183.0862	69.0337<br>57.0330	+	+
**D-Mannitol 1-phosphate**	261.0379	96.9694<br>78.9591	+	+
**Sedoheptulose**	209.0664	59.0128<br>57.0335	+	+
***α*-D-Glucose**	179.0556	71.0141<br>59.0134	+	+
**Sugar acids and derivatives**				
**Galactonic acid**	195.0511	75.0090<br>59.0139	+	+
**Glucuronic acid**	193.0349	75.0088<br>59.0139	+	+
**Glyceric acid**	105.0189	59.0143<br>56.9850	+	+
**2-Dehydro-3-deoxy-L-arabinonate**	147.0300	85.0284<br>57.0335	+	+
**Glycerol-2-phosphate**	171.0066	96.9688	+	+

		78.9594		
**Xylonate**	165.0402	75.0089<br>59.0140	+	+
**Fatty acids and conjugates**				
**2-Hydroxyisocaproic acid**	131.0715	85.0660<br>69.0340	+	+
**3-Hydroxycapric acid**	187.1341	59.0138<br>41.0031	-	+
**6,7-Epoxystearic acid**	297.2438	297.2427<br>185.1178	-	+
**9,10-Dihydroxystearic acid**	315.2541	298.2502<br>44.9971	-	+
**9,10-Epoxy-18-hydroxystereate**	313.2382	113.0961<br>99.0804	-	+
**Epsilcapramine**	132.1020	59.0724<br>132.1018	+	+
**Stearic acid**	283.2646	283.2638	-	+
***α*-Licanic acid**	293.2089	116.0505<br>45.0336	-	+
**Fatty amides**				
**13-Docosenamide**	338.3418	83.0855<br>57.0697	-	+
**Oleamide**	282.2793	71.0861<br>57.0693	-	+
**Sphingolipids**				
**C16 Sphinganine**	274.2739	256.2622<br>106.0853	-	+
**Sphinganine**	302.3058	284.2943<br>57.0692	-	+
**Phospholipid and derivative**				
**Phosphocholine**	184.0731	124.9997<br>86.0967	+	+
**Glycerophosphocholine**	258.1099	124.9996<br>104.1070	+	+
**No metal oxoanionic compounds**				
**Phosphate**	96.9694	96.9690<br>78.9594	+	+
**Phosphite**	78.9588	78.9599<br>62.9646	+	+
**Phenol derivative**				
**p-Aminophenol**	110.0601	93.0336<br>65.0387	-	+
**Purine nucleoside derivative**				
**Xanthosine**	283.0650	151.0260	+	+
**Purine nucleotides and derivatives**				
**3´-AMP**	346.0559	211.0014<br>78.9590	+	+
**AMP**	348.0702	136.0619<br>97.0285	+	+
**ADP**	426.0220	158.9250<br>78.9593	+	+
**cGMP**	344.0405	150.0421	+	+

		108.0203		
**GDP**	442.0173	344.0402<br>150.0421	+	+
**GDP-mannose**	604.0701	442.0177<br>424.0057	+	+
**GMP**	364.0654	152.0567<br>97.0289	+	+
**N6-(1,2-Dicarboxyethyl)-AMP**	464.0814	252.0724<br>234.0620	+	+
**Purines and purine derivatives**				
**Adenine**	134.0469	107.0360<br>92.0248	+	+
**Guanine**	152.0568	135.0302<br>110.0351	+	+
**Hypoxanthine**	137.0454	119.0351<br>110.0349	+	+
**Uric acid**	167.0210	124.0154<br>41.9985	+	+
**Xanthine**	151.0260	108.0196<br>41.9987	+	+
**Pyrimidine nucleotides and derivatives**				
**UMP**	323.0291	96.9691<br>78.9592	+	+
**cUMP**	305.0183	111.0197<br>41.9985	+	+
**UDP-D-xylose**	535.0374	323.0286<br>78.9591	+	+
**UDP-glucose**	565.0479	384.9842<br>323.0289	+	+
**UDP-glucuronic acid**	579.0271	402.9954<br>323.0289	+	+
**UDP-N-acetylglucosamine**	606.0744	384.9850<br>282.0392	+	+
**Pyrimidine derivative**				
**Uracil**	111.0199	41.9988	+	+
**Pyridine derivative**				
**Nicotinic acid**	124.0394	80.0497<br>78.0335	+	+

[1]MS: Mass spectrometry

Table S4. Percentage of LX2 cells death using different concentrations of AB mushroom extract.

AB concentration (mg/mL)	Total cell count	Viable cell count	Death cell Count	Viability (%)	Cell death (%)
10	14.8 x 10^6	1.8 x 10^6	13.0 x 10^6	12.16	87.83
5	18.2 x 10^6	3.6 x 10^6	14.6 x 10^6	19.78	80.21
2	23.8 x 10^6	13.6 x 10^6	10.2 x 10^6	57.14	42.85
1	25.6 x 10^6	23.2 x 10^6	2.4 x 10^6	90.62	9.37
0.2	27.6 x 10^6	26.3 x 10^6	1.3 x 10^6	95.28	4.71

Table S5. Macronutrient composition of experimental diets.

	LFD control	LFD + AB	HFD45	HFD60 + AB
Energetic content (kJ/g)	11.8	11.8	15.7	21.0
Carbohydrates (w/w)	48	48	39	39
Proteins (w/w)	14	14	12	12
Fats (w/w)	4	4	19	33
Colesterol (mg/kg)	-	-	98	98
Mushroom extract*	0	7.5	0	7.5
**Total caloric intake**				
Carbohydrate (kcal%)	67.6	67.6	41.6	31.1
Proteins (kcal%)	19.7	19.7	12.8	9.6
Fats (kcal%)	12.7	12.7	45.6	59.3

*The AB mushroom extract was supplemented in the water, adding 7.5 g/kg mouse in the feeding bottle three times per week.

Table S6.a. Distribution of control and treated groups for acute toxicity study in rats. ACT-0= Acute toxicity control group treated with distilled water (3 mL). ACT-I/ II / III: Acute toxicity group with a dose of 300 (I) / 2000 (II) / 5000 (III) mg/kg BW of AB extract.

Group:	ACT-0	ACT-I	ACT-II	ACT-III
**n**	10	10	10	10
**Male**	5	5	5	5
**Female**	5	5	5	5
**Dose (mg/kg)**	0	300	2000	5000

Table S6.b. Acute toxicity study in rats (dose 5000 mg/kg BW of AB extract).

Observation	After AB			
	4h	8h	24h	14 days
**Skin and fur**	Normal	Normal	Normal	Normal
**Eye**	Normal	Normal	Normal	Normal
**Mucous membrane**	Normal	Normal	Normal	Normal
**Behavioral patterns**	Normal	Normal	Normal	Normal
**Salivation**	Normal	Normal	Normal	Normal
**Lethargy**	Normal	Normal	Normal	Normal
**Diarrheal**	No observed	No observed	No observed	No observed
**Tremors**	No observed	No observed	No observed	No observed
**Coma**	No observed	No observed	No observed	No observed
**Deaths**	No observed	No observed	No observed	No observed

Table S7. List of specific human primers.

Gene	Cat.no	Commercial home
GAPDH	QT00079247	QIAGEN
COL1α1	QT00037793	QIAGEN
ACTIN-2α	QT00088102	QIAGEN
TPM2β	QT00048720	QIAGEN
GATA4	QT00031997	QIAGEN
SOD1	QT01008693	QIAGEN
iNOS	QT00068740	QIAGEN

Table S8. List of specific mouse primers.

Gene	Cat.no	Commercial home
GADPH	QT01658692	QIAGEN
COL1α1	QT00162204	QIAGEN
GATA4	QT00155400	QIAGEN
SOD1	QT00161707	QIAGEN
iNOS	4331182	TaqMan
TLR4	Mm00445273_m1	TaqMan
PPARα	Mm00440939_m1	TaqMan
TNFα	Mm00443258_m1	TaqMan
GPX3	Mm00492427_m1	TaqMan

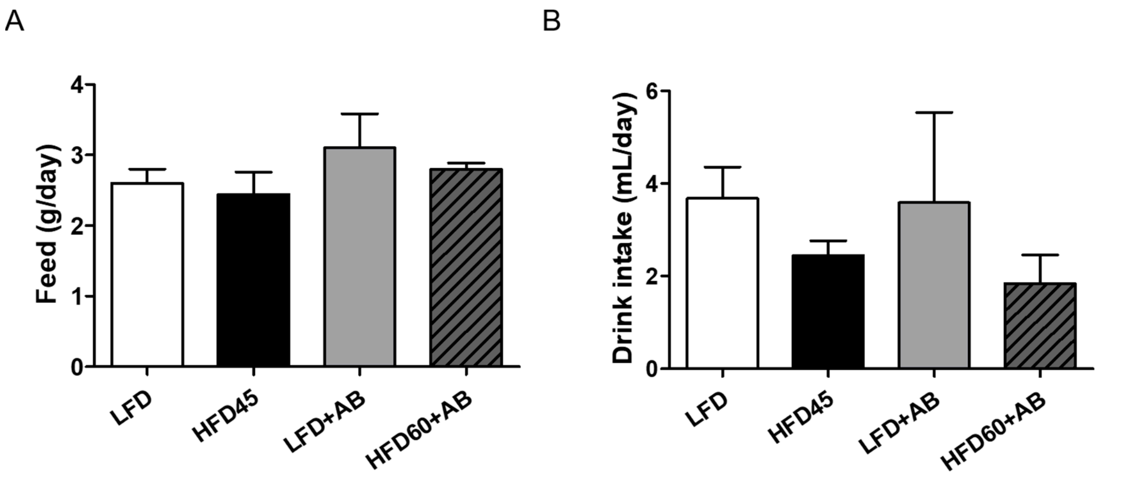

Figure S1. Indirect calorimetric analyses of A) Average food intake mouse/day and B) Average drink intake mouse/day. All data are shown as means ± SD (n= 4). No significant differences were observed among groups.

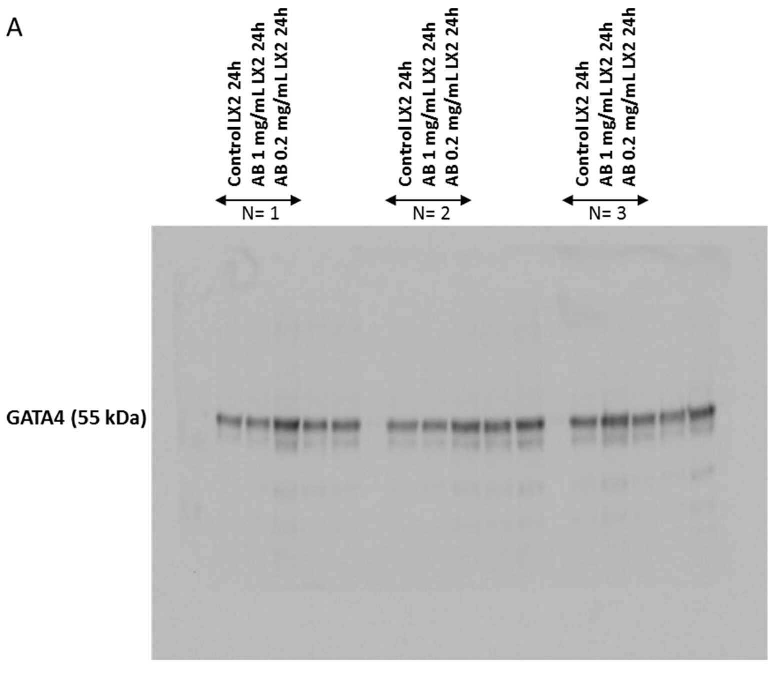

B

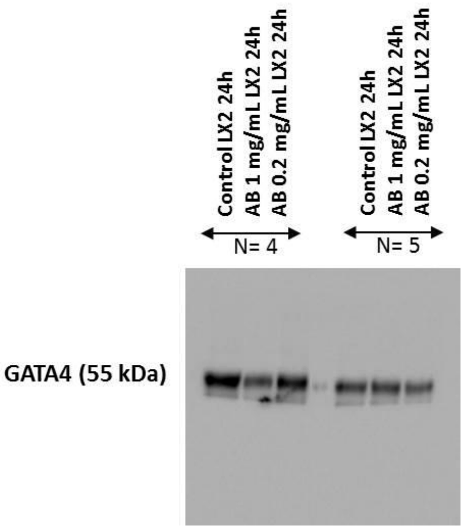
Control LX2 24h
AB 1 mg/mL LX2 24h
AB 0.2 mg/mL LX2 24h
Control LX2 24h
AB 1 mg/mL LX2 24h
AB 0.2 mg/mL LX2 24h
N= 4
N= 5
GATA4 (55 kDa)

C

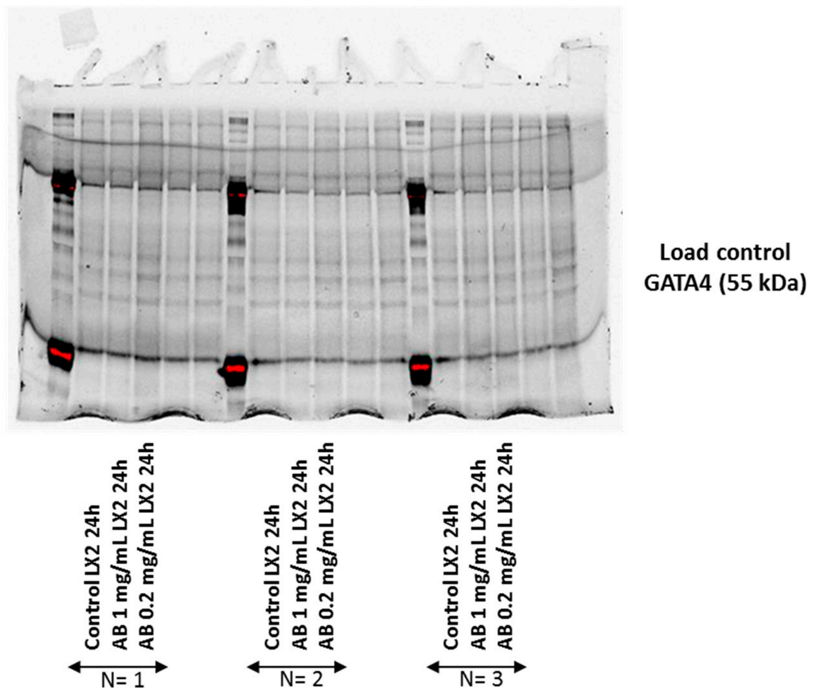
Load control
GATA4 (55 kDa)
Control LX2 24h
AB 1 mg/mL LX2 24h
AB 0.2 mg/mL LX2 24h
Control LX2 24h
AB 1 mg/mL LX2 24h
AB 0.2 mg/mL LX2 24h
Control LX2 24h
AB 1 mg/mL LX2 24h
AB 0.2 mg/mL LX2 24h
N= 1
N= 2
N= 3

D

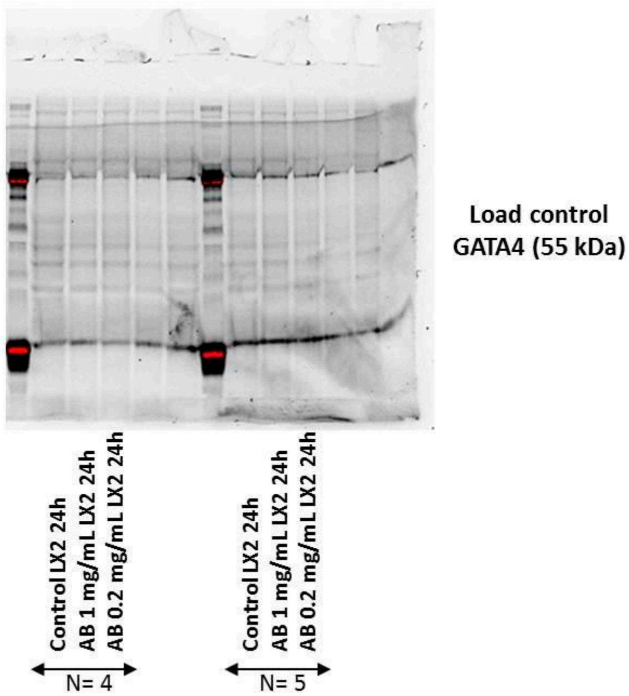

Figure S2. Blot images (*n*= 5) of protein expression experiments in LX2 cells treated with AB extracts at 0.2 mg/mL and 1 mg/mL for 24h. (A) and (B) GATA4. (C) and (D) Load Control GATA4. Blot B is a cut of a complete membrane whose size is 13 x 7.5 cm. Bands corresponding to figure S2A (experiment number 2) and figure S2C (experiment number 2) were picked as representative images in figure 1F.

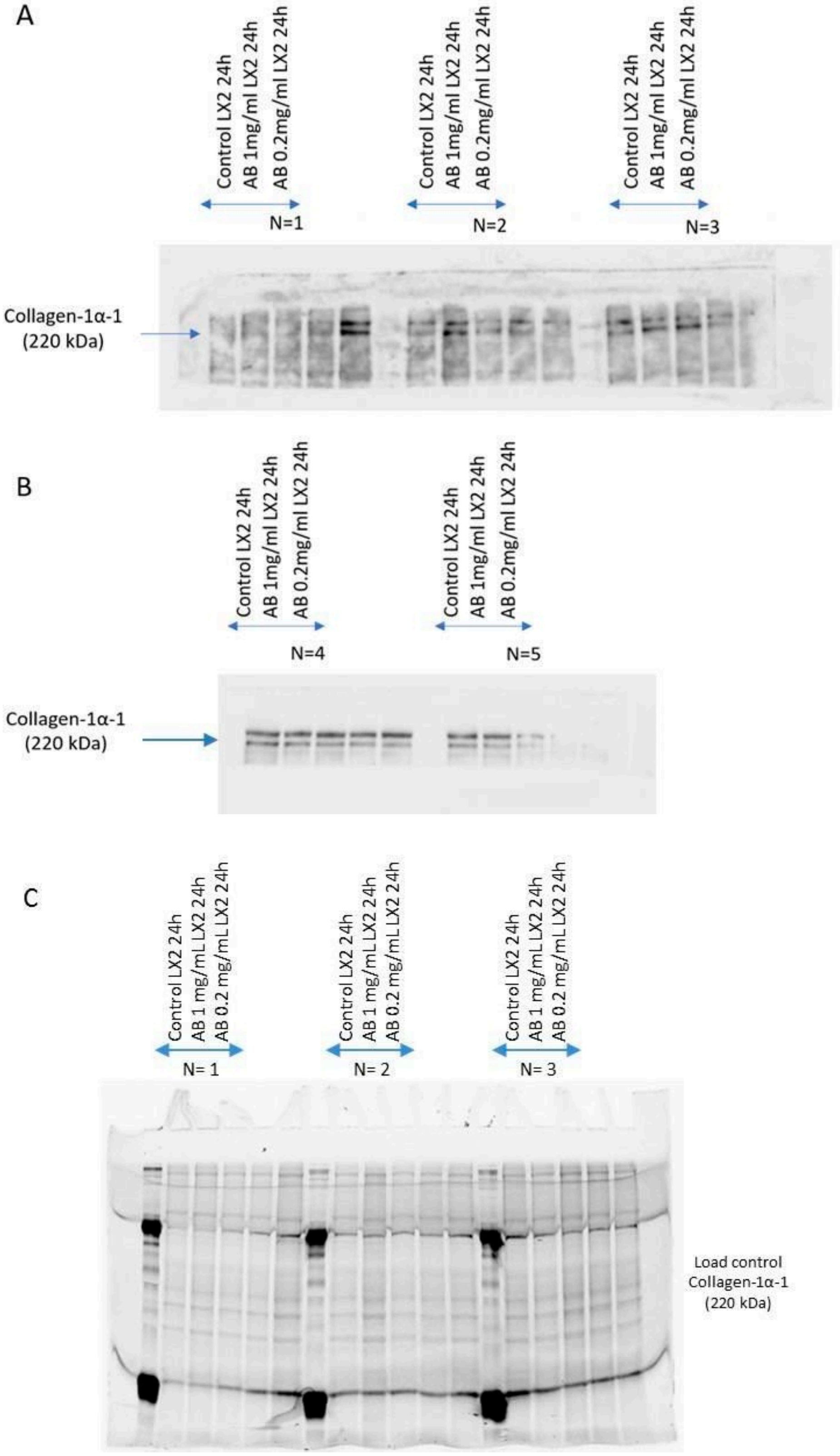
A
Control LX2 24h
AB 1mg/ml LX2 24h
AB 0.2mg/ml LX2 24h
N=1
N=2
N=3
Collagen-1α-1
(220 kDa)
B
Control LX2 24h
AB 1mg/ml LX2 24h
AB 0.2mg/ml LX2 24h
N=4
N=5
Collagen-1α-1
(220 kDa)
C
Control LX2 24h
AB 1 mg/mL LX2 24h
AB 0.2 mg/mL LX2 24h
N= 1
N= 2
N= 3
Load control
Collagen-1α-1
(220 kDa)

D

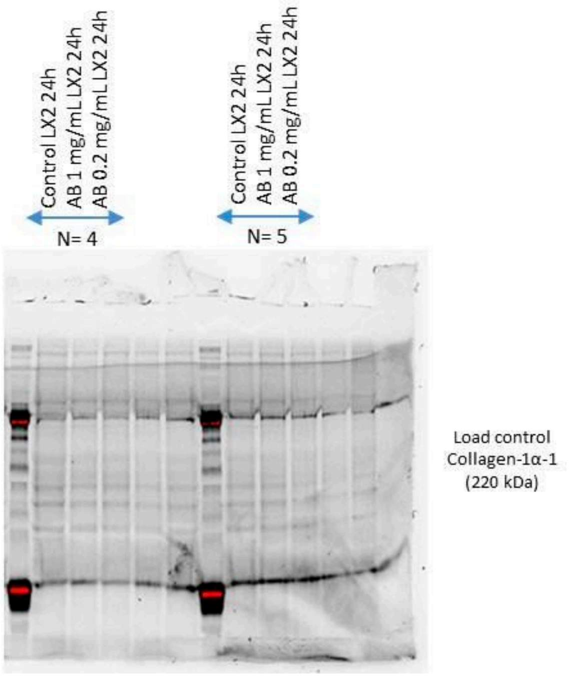

Figure S3. Blot images (*n*= 5) of protein expression experiments in LX2 cells treated with AB extracts at 0.2 mg/mL and 1 mg/mL for 24h. (A) and (B) COL1α1. (C) and (D) Load Control COL1α1. Blots A and B are cuttings of a complete membrane whose size is 13 x 7.5 cm. Bands corresponding to figure S3A (experiment number 2) and figure S3C (experiment number 2) were picked as representative images in figure 1FD
